# Interaction with refuse piles is associated with co-occurrence of core gut microbiota in workers of the ant Aphaenogaster picea

**DOI:** 10.1099/acmi.0.000832.v4

**Published:** 2025-01-30

**Authors:** Alison Pagalilauan, Christina Pavloudi, Santiago Meneses Ospina, Adam Smith, Jimmy H. Saw

**Affiliations:** 1 Department of Biological Sciences, The George Washington University, Washington DC 20052, USA; 2 European Marine Biological Resource Centre-European Research Infrastructure Consortium (EMBRC-ERIC), Paris, France; 3 Department of Biological Sciences, New Jersey Institute of Technology, Newark, NJ 07102, USA

**Keywords:** *Aphaenogaster*, bacterial community, colony phenotype, division of labour, microbiome, social insects, task specialization, 16S rRNA

## Abstract

Comparing the diversity of gut microbiota between and within social insect colonies can illustrate interactions between bacterial community composition and host behaviour. In many eusocial insect species, different workers exhibit different task behaviours. Evidence of compositional differences between core microbiota in different worker types could suggest a microbial association with the division of labour among workers. Here, we present the core microbiota of *Aphaenogaster picea* ant workers with different task behaviours. The genus *Aphaenogaster* is abundant worldwide, yet the associated microbiota of this group is unstudied. Bacterial communities from *Aphaenogaster picea* gut samples in this study consist of 19 phyla, dominated by Proteobacteria, Cyanobacteria and Firmicutes. Analysis of 16S rRNA gene sequences reveals distinct similarity clustering of *Aphaenogaster picea* gut bacterial communities in workers that have more interactions with the refuse piles. Though gut bacterial communities of nurse and foraging ants are similar in overall composition and structure, the worker groups differ in relative abundances of dominant taxa. Gut bacterial communities from ants that have more interactions with refuse piles are dominated by amplicon sequence variants associated with Entomoplasmataceae. Interaction with faecal matter via refuse piles seems to have the greatest impact on microbial taxa distribution, and this effect appears to be independent of worker type. This is the first report surveying the gut microbiome community composition of *Aphaenogaster* ants.

## Data Summary

Raw sequence files generated in this study have been deposited in the Sequence Read Archive (SRA) database at NCBI under the BioProject ID PRJNA1076551. The complete list of SRA accession numbers of the raw sequences is shown in Table S1 in the data supplement. All supplemental materials and video data are publicly available on Figshare (https://doi.org/10.6084/m9.figshare.25546411.v2). The R code and workflow used in the study are available on the following GitHub repository: https://github.com/pagalila1/aphaenogaster-16S.

## Introduction

Associations between behavioural specificity and gut microbiome composition present an opportunity to study whether microbiomes are associated with different worker tasks in a social insect colony [1,4]. In social insect colonies, a queen reproduces and workers do not. In many social insect groups, there are behavioural subcastes among the workers: some workers perform extranidal (outside the nest) tasks such as foraging and patrolling, while others perform intranidal (inside the nest) tasks such as brood care and nest maintenance [5]. Individuals serving different roles within a colony might benefit from symbiotic relationships that augment the different metabolic processes necessary for those roles. Additionally, workers that perform particular tasks may acquire different microbes, for instance, as a result of foraging outside the nest versus working inside [4,6]. In either case, the division of labour among the workers may lead to differences in gut microbiota between behavioural castes.

Gut microbiota studies are powerful for understanding the role of symbionts in promoting host dynamics. For example, the bacterial genus *Lactobacillus* is commonly involved in promoting complex carbohydrate processing in a wide range of organisms and can be found in high abundance in honeybees performing nest tasks such as brood care and food processing [4,6]. In honeybees (*Apis mellifera*), the microbiota is linked to colony and individual health [7,10]. Disruption of microbiota leads to increased disease [8]. The microbial composition differs between nurse and forager subcastes of workers [6,10, 11], and foragers have a lower diversity of core gut bacteria, potentially making them more susceptible to invasion by new strains [10]. Moreover, gut microbiota may influence the neurobiology and physiology that underlie subcaste behavioural differences [12].

Symbioses between microbiota and social insects exist in ants as well. The gut environment of nurses and other intranidal workers is especially adapted to breaking down macronutrients in the guts of herbivorous turtle ants (*Cephalotes*) [13,16]. In the case of both turtle ants and honeybees, nutritional mutualisms between bacteria and task-specialized workers may augment task specialization and maximize colony fitness [17,18]. In further support of task-associated microbiome assemblage, another study determined that functionally distinct *Azteca* ant nest chambers maintain unique microbial communities. Nursery nest chambers show particularly low diversity [19]; these observations suggest that nurses may increase colony productivity by inhibiting bacteria that could compromise larval survival. Alternatively, bacteria belonging to the phylum Actinobacteria may produce antibiotics or otherwise increase immunity in nurses, as is the case with leaf-cutting ants [20]. These studies highlight ways in which behavioural castes may have distinct microbiomes associated with behavioural roles. Nevertheless, while there are examples of differences in the microbiota of the reproductive caste (queens and males) and the worker caste in ants [21,24], there is little evidence of microbiota differences between the nurse and forager subcastes of workers [25,26].

Workers of *Aphaenogaster picea* do not have morphological subcastes, but some individuals behaviourally specialize on specific tasks, while others indiscriminately perform all task types. The two general worker tasks that we focus on in this study are feeding larvae (nurse workers) and foraging outside the nest for food (foragers). Generalist workers that both feed larvae and forage may have a greater need to maintain immunity [27], given their frequent interactions with multiple environments. Should such generalist workers benefit from bacteria that aid in carbohydrate processing or antibiotic secretion, increased microbiome diversity or abundance would be expected when compared to specialized workers, although the coevolution of microbiota specialized for individual subcastes is likely constrained by uniform inheritance from the queen. In contrast, the core microbiota of specialized *Aphaenogaster* workers (i.e. nurses and foragers) may reflect the intranidal or extranidal environments that they are predominantly interacting with, leading to less diverse bacterial communities than generalists that may acquire microbiota from both inside and outside the nest. This has been observed in *Azteca* ant colonies, where the composition of the ant microbiome mirrors that of the surrounding soil and particular nest chamber inhabited by each individual ant [19,28]. Though laboratory conditions rarely capture the complete range of bacterial diversity that may exist in field settings, termite nest-associated microbiomes have been shown to differ from termite-associated microbiomes in a laboratory setting [29]. Acidobacteria were twice as abundant in an infected termite nest than in uninfected nests [29]. Bacterial taxa such as Acidobacteria, which are ubiquitous in most soil communities, may be abundant in *Aphaenogaster* workers that spend more time inside the nest [30].

*Aphaenogaster picea* is a generalist that scavenges other insects and eats the nutritious elaiosomes that some plants attach to seeds in order to encourage dispersal by ants [31]. In other species, specialized scavenging can lead to gut microbiome specializations that reduce the risk of diseases that may arise from consuming decaying tissue, pathogenic bacteria and faecal matter [32]. Like many ant species, *Aphaenogaster picea* are omnivorous. Previous studies of the microbiota of ants have focused on herbivorous species such as *Cephalotes* [13,33, 34], as well as carnivorous and herbivorous ants [35,36]. In a previous microbiome study of omnivorous weaver ants, bacterial communities were dominated by plant-associated taxa despite host predation on other arthropods [37]. In a microbial community study of ants with different feeding strategies, bacterial communities differed between carnivorous and herbivorous ants and herbivorous and omnivorous ants, but not between carnivorous and omnivorous ants [38]. In such studies, surveying the microbiome of omnivorous species can show how different feeding strategies lead to differences in gut bacterial community assemblage.

Although the microbiome of any species of *Aphaenogaster* has never been characterized, experimental manipulations with antibiotics can impact host function. Antibiotic treatments alter cuticular hydrocarbon profiles and reduce alkaloid secretion in *Aphaenogaster* species with poison glands, possibly by disrupting microbial symbioses [39]. In response to the antibiotic treatment of surface-dwelling symbionts in a similar study, *Camponotus* ants experienced an increase in cuticular hydrocarbons, suggesting that the removal of core symbionts triggers an immune response to protect the host against desiccation [40]. While these investigations have only focused on cuticle microbiota, such drastic host effects emphasize the potential functional importance of the *Aphaenogaster* gut bacterial community.

The aim of this investigation is to, first, describe the microbiota of *Aphaenogaster picea* ants. Second, we compare gut microbiota between worker subcastes of *Aphaenogaster picea*. Many social insects share food through trophallaxis, the transfer of fluid food between individuals [41]. This can have the effect of homogenizing gut bacterial composition among colony members [41]. *Aphaenogaster* ants do not engage in trophallaxis; thus, their gut microbial communities are less likely to be homogenous. Some ant species, such as *Cephalotes rohweri*, which do perform trophallaxis*,* have a specialized proventriculus that acts as a filter to selectively allow bacteria to enter the midgut [35]. *Aphaenogaster* ants do not have such a modified proventriculus. Because they do not perform trophallaxis or have proventricular filters, we predict that there will be differences in gut microbial communities between individuals in the same colony. More specifically, we predict that there will be higher bacterial abundance and diversity in the guts of generalists than those of specialized workers. Many other social insects have worker division of labour similar to *Aphaenogaster* in which morphologically similar workers vary in their propensity to undertake different tasks [42]. Thus, identifying task-associated differences in gut microbiome composition among the weakly defined subcastes of *Aphaenogaster* would imply a potentially widespread pattern in other social insects with behaviourally specialized workers.

## Methods

### Colony care and observations

Colonies of *Aphaenogaster picea* were collected in the summer (July–August 2021) at 900–1000 masl in the vicinity of lat. 38.437 and long. -78.478 in Greene and Page Counties, VA, USA. Colonies were identified to species using the key provided in [43]. *Aphaenogaster picea* are more common in higher elevations (above 900 masl) and are distinguishable from the common *Aphaenogaster rudis* by lighter colouration in the last four segments of the antennae [43]. Vouchers were deposited in the Smithsonian insect collection, Washington, DC, USA. Collected colonies were kept under controlled laboratory conditions at George Washington University, Washington, DC, USA, for the duration of this study. Each colony consisted of 100–300 workers. Colonies were fed an artificial diet made from 300 ml distilled water, 4.23 g egg powder, 5.22 g whey protein, 5.85 g calcium caseinate, 14.7 g sucrose, 0.81 g Vanderzant vitamin mixture (*α*-tocopherol 8 g kg^−1^, ascorbic acid 270 g kg^−1^, biotin 20 mg kg^−1^, calcium pantothenate 1 g kg^−1^, choline chloride 50 g kg^−1^, crystalline folic acid 250 mg kg^−1^, inositol 20 g kg^−1^, niacinamide 1 g kg^−1^, pyridoxine hydrochloride 250 mg kg^−1^, riboflavin 500 mg kg^−1^, thiamine hydrochloride 250 mg kg^−1^ and vitamin B12 trituration in mannitol 2 g kg^−1^ Q.S. with dextrose) and 4.8 g agar. Diets were replaced every 24–36 h, and each colony was additionally supplemented with sucrose water. Colonies were stored in 50–80% humidity at 20 °C with constant light exposure. Colonies were subjected to fixed conditions to disrupt temporal hibernation cues associated with changes in light intensity, humidity and temperature.

**Fig. 1.**
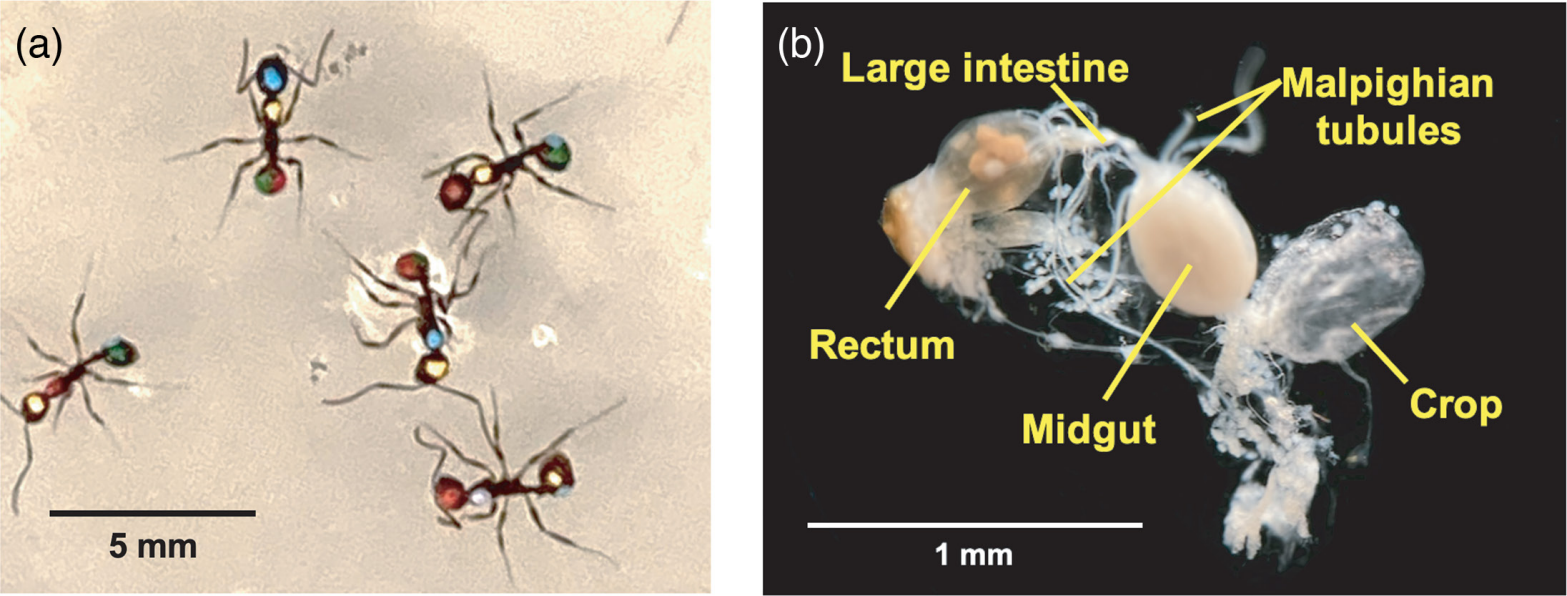
(a) Paint-marked workers. Ants were labelled with paint in four positions: one mark on the head (below the eyes), one mark on the thorax (pronotum) and two marks on the gaster (first segment, left and right side). (b) Gut sample isolated from *Aphaenogaster picea* cuticle. The image shows a dorsal view of the digestive tract with the distal end of the gaster on the left side. The large intestine and rectum comprise the hindgut. Malpighian tubules closely surround the midgut prior to dissection.

Only colonies with healthy queens and observable larvae were sampled in this study. All individuals in each colony were marked with Testors (Rockford, IL, USA) enamel paint unique colour permutations to distinguish each worker (Fig. 1a). Paint-marked workers were observed over the span of 3–4 days. Behavioural data were collected by video recordings and real-time observations. Foragers were identified as individuals that demonstrated increased interaction with food sources and/or overall extranidal activity, and nurses were identified as individuals that demonstrated increased interaction with larvae and/or overall intranidal activity. Individuals that were equally active inside and outside the nest, or otherwise equally participated in foraging and brood care, were categorized as generalist workers.

Two 30-min observation periods were conducted on separate days to collect behavioural data for each colony. Data were manually recorded from behaviour observed in videos. The ratio of time spent outside the nest to total time observed was used as the primary identifier of forager, nurse or generalist status (Table S1, available in the online Supplementary Material). The amount of time that each worker spent outside the nest was used to define forager status, as leaving the nest is associated with foraging and patrolling. Inversely, the amount of time that each worker spent inside the nest was used to define nurse status, as staying inside the nest is associated with nursing and nest care. % Extranidal (%E) scores were calculated by dividing the amount of time that each individual spent outside the nest by the total time observed and multiplying the score by 100. Nurses were designated as individuals with scores less than 25%, generalists were designated as individuals with scores between 25 and 75% and foragers were designated as individuals with scores greater than 75%. Three markers of task behaviour were recorded during colony observations: intranidal movement, foraging activity and faecal interaction. Intranidal movement and foraging activity were measured to determine whether increased general tempo had any effect on worker microbiota diversity or abundance. Intranidal movement was measured as the total number of body lengths travelled by an individual whenever inside the nest. Foraging activity was measured as the number of instances that each individual interacted with liquid or solid diet materials in the laboratory habitat. Faecal interactions through physical contact with debris (e.g. dead ants, decaying food and faecal matter) were measured as the number of instances that each individual interacted with refuse piles. Each behavioural measurement was divided by the amount of time that each individual was observed and then reported as a ratio (Table S1). Intranidal movement score (IMS), foraging score (FoS) and faecal interaction score (FeS) were each calculated as the number of instances of that behaviour divided by the total time observed per worker. Score ranges were used to categorize each individual’s level of activity with respect to each behavioural variable (Table 1).

**Table 1.** Intranidal and extranidal activity score range. Discrete variables were assigned to each range for IMS, FoS and FeS. See Table S1 for numerical activity data

IMS		FoS		FeS	
Very high	>10.0	High	>0.75	High	>0.75
High	5.0–10.0	Intermediate	0.26–0.75	Intermediate	0.26–0.75
Intermediate	1.0–4.99	Low	0.01–0.25	Low	0.0–0.25
Low	<1.0	None	0		

### Tissue dissection and DNA extraction

To prepare samples for tissue dissections and DNA extractions, individuals were grouped by foraging behaviour within each colony. The total sample size (*n*=50) was sourced from three *Aphaenogaster picea* colonies, and workers of each task group were isolated for dissection of the gaster and legs. More specifically, 20 individuals were sampled from colony A48, 10 individuals were sampled from colony A51 and 20 individuals were sampled from colony A53. Samples were isolated from colonies immediately after the video collection of behavioural data. To maximize sampling of gut-colonizing bacteria, gut samples were separated from the cuticle of the gaster during dissection (Fig. 1b).

Samples were stored in RNAlater (Thermo Fisher Scientific) at 4 °C prior to DNA extraction. Samples were diluted in equal parts nuclease-free water and then centrifuged at 11 000 ***g*** for 8 min to dissolve any RNAlater precipitates. The supernatant was discarded before sample pellets were homogenized by manual grinding in tissue lysis buffer. DNA was extracted using the Qiagen Blood and Tissue Kit. Extractions were performed per the manufacturer’s instructions with an extended incubation time for sample lysis. Samples were incubated in tissue lysis buffer with Proteinase K at 56 °C for 24 h. A reduced elution volume of 20 µl was used to maximize total DNA yield. Total DNA yield per sample was quantified by Qubit 4 Fluorometer (Thermo Fisher Scientific). Before sequencing, DNA quality was assessed by PCR amplification of primers 515F [44] and U1391R [45] and visualization of PCR products with 1% agarose gel electrophoresis.

### 16S rRNA amplicon sequencing

Though leg dissections were collected, DNA extractions from homogenized legs did not yield adequate concentrations for downstream analyses and were thus omitted from 16S rRNA amplicon sequencing. To assess differences in species abundance and diversity across gut compartments of forager and nurse worker subcastes, DNA isolated from each gut sample was submitted to the ASGPB Sequencing Lab at the University of Hawaiʻi at Mānoa for 16S rRNA amplicon sequencing. The V3–V4 regions of the bacterial 16S rRNA gene were amplified with the primers S-d-Bact-0341-b-S-17 (5′-CCTACGGGNGGCWGCAG-3′) and S-d-Bact-0785-a-A-21 (5′-GACTACHVGGGTATCTAATCC-3′) [46]. Amplicons were sequenced on the Illumina MiSeq platform (using Reagent Kit v3) in a 600-cycle (2×300 bp) paired-end run.

To analyse bacterial sequence data, DADA2 (v1.14) [47] was used to identify amplicon sequence variants (ASVs). Raw sequences were trimmed using the following parameters: maxEE=c(5,8) and truncQ=0; the trimRight parameter was removed from this analysis. Taxonomic assignment was conducted against the silva (v138.1) reference database [48].

### Statistical analysis

All statistical analyses were conducted using R (v4.2.1) [49]. Decontamination was performed in the ASV table, based on the prevalence of ASVs in the negative (blank) control using the decontam package (v1.14.0) [50]. One-way ANOVA and Kruskal–Wallis tests were conducted to compare IMS, FoS and FeS measurements between each task group. Alpha diversity [abundance coverage estimator (ACE), Chao1 and Shannon] indices were also analysed by one-way ANOVA to compare the gut microbiota of workers. To assess beta diversity of gut bacterial communities, we performed non-metric multidimensional scaling (NMDS) analysis, based on the Bray–Curtis dissimilarity between gut bacterial communities, and principal coordinate analysis (PCoA), based on UniFrac distances. NMDS clustering was analysed for significance by conducting permutational multivariate ANOVA (PERMANOVA). Network representations of samples were generated using the Jaccard dissimilarity index and a maximum distance between connected nodes of 0.4. All the aforementioned alpha and beta diversity analyses were conducted with phyloseq (v1.40.0) [51] and vegan (v2.6.2) [52] packages. Using phyloseq, a phylogenetic tree was constructed from ASVs belonging to the phylum Acidobacteria to investigate the potential for host specificity between bacterial genera and worker type. Taxonomic groups that had significant differences in abundance among different colonies or groups (e.g. ‘forager’ vs ‘nurse’) were identified by linear discriminant analysis effect size (LEfSe) analysis [53] using the microbiomeMarker package (v1.3.2) [54]. Upset plots showing how many ASVs and how many species were unique and how many were shared between the different groups were also generated using the UpSetR (v1.4.0) [55] and ComplexUpset (v1.3.3) [56,57] packages. R packages ggplot2 (v3.3.6) [58] and ggpubr (v0.4.0) [59] were used for the aforementioned visualizations.

## Results

### Behavioural observations

Of the 50 workers sampled, a majority were identified as nurses (*n*=22) or foragers (*n*=22). The few workers demonstrating generalist (*n*=6) behaviour did not differ from specialist workers in how they performed nursing and foraging behaviour. We expected generalists to interact with food and brood with equal frequency. Under this assumption, we expected to observe simultaneously high FoS (foraging) and IMS (intranidal movement) in generalists, in addition to %E (extranidal) scores between 25 and 75%. However, we did not observe high scores in both FoS and IMS in workers designated as generalists by %E scoring. In the absence of significant differences in these measures of behaviour between generalists and other worker types, the six generalists were pooled into the larger nurse and forager groups for the analysis of 16S rRNA sequencing data. Generalists with %E scores less than 50% were considered nurses (*n*=1), and generalists with %E scores greater than 50% were considered foragers (*n*=5) (Table S1).

Each colony typically maintained an intranidal refuse pile along one side of the nest and one or two extranidal piles in the corners of the habitat (Fig. S1). Both intranidal and extranidal individuals interacted with refuse piles, but there was no significant correlation between either worker type and FeS. Nurses with low IMS were less likely to interact with refuse piles, but foragers did not have significantly higher FeS than nurses overall. Anecdotally, we noticed that generalists and foragers seemed to interact more with extranidal waste piles especially right before being fed (the point at which food was most scarce), but our recordings did not include this period. There was no significant correlation between foraging and IMS or nursing and IMS. As expected, given the definition of the groups, there was a significant difference in FoS between groups was confirmed by ANOVA (Table 2).

**Table 2.** ANOVA results comparing variation of IDS, FoS and FeS between all worker groups

Variable	SS	MS	F	***P*-value**
IDS	19.84	9.92	0.20	0.82
FoS	2.05	1.03	21.12	2.86E−07
FeS	0.37	0.18	1.31	0.28

* Significant variation between groups (df=2, *F*-crit=3.195).

FF-valueMSMean squaresSSSum-of-squares

### Microbiome community composition

**Fig. 2.**
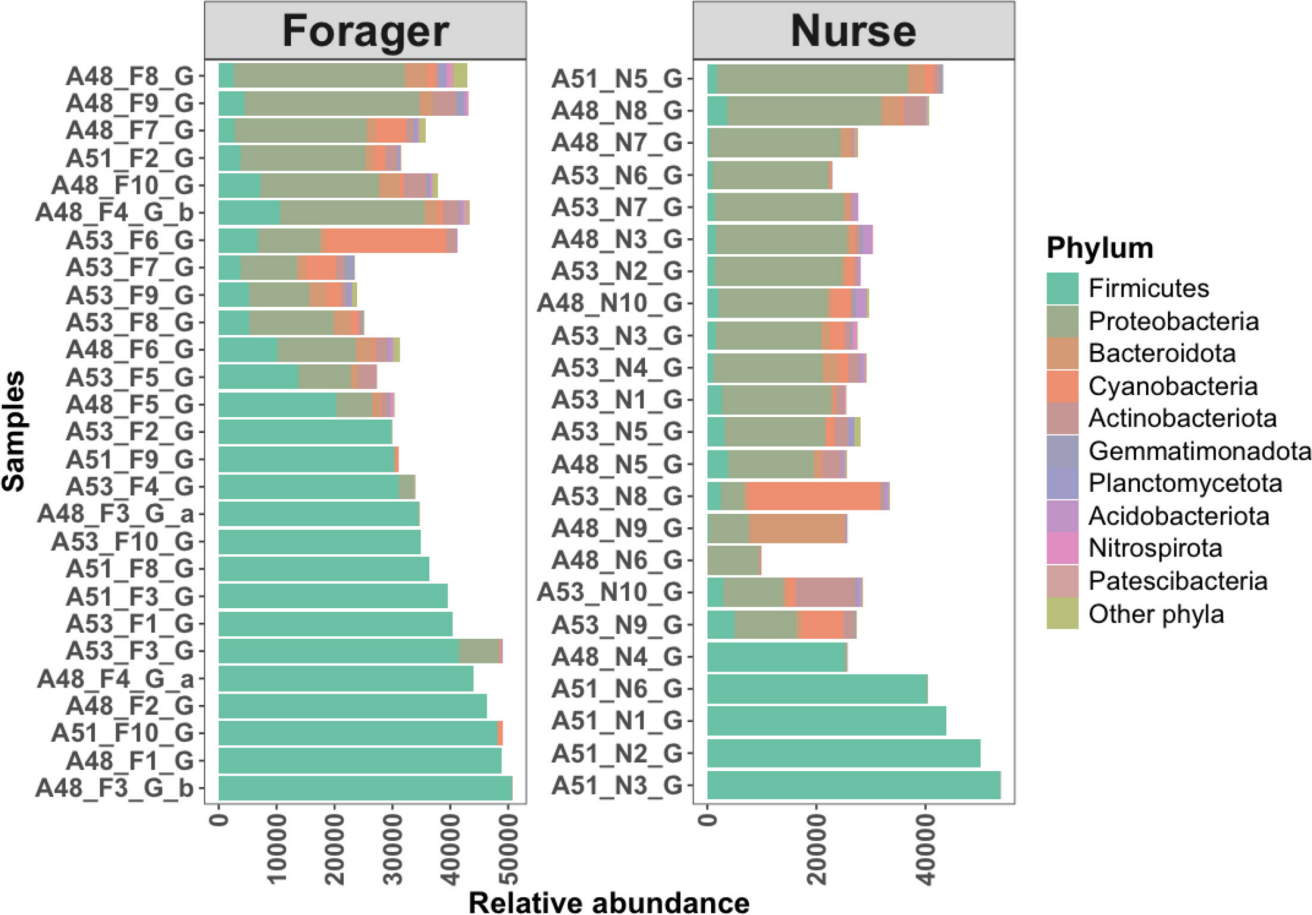
Relative abundance of bacterial phyla per gut sample community. The plot is split into two subplots, showing the abundance of taxa in foraging (left) and nurse (right) ants. Each colour represents different taxa identified in the sample sequences.

**Fig. 3.**
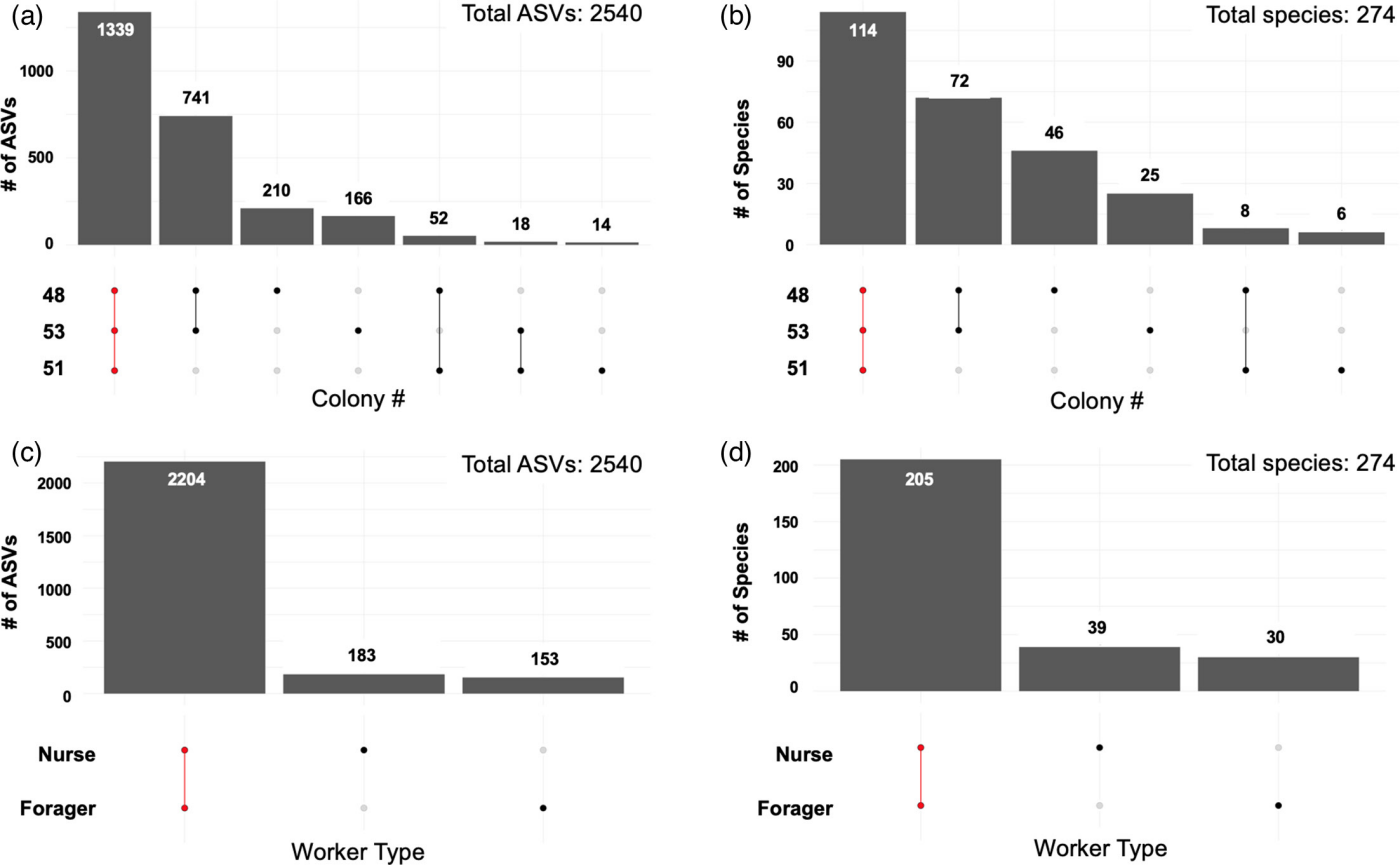
Upset plots showing the number of ASVs (**a, c**) and species (**b, d**) that were unique or shared between the different groups. Darkened circles below each bar denote ASVs or species detected from samples of a specific colony or worker type. The red dotted bars represent ASVs or species detected in samples from all colonies (**a, b**) or samples from all worker types (**c, d**).

Decontamination resulted in the removal of 154 ASVs from the ASV table, almost exclusively belonging to *Pseudomonas*. 16S rRNA sequencing of gut samples taken from 50 *Aphaenogaster picea* workers revealed 5 dominating core phyla: Firmicutes (Bacillota), Proteobacteria (Pseudomonadota), Cyanobacteria (Cyanobacteriota), Bacteroidetes (Bacteroidota) and Actinobacteria (Actinomycetota) (Table 3, Fig. 2 and S1). The remaining taxa contributed to 2.4% of all ASVs. A total of 19 phyla were identified from the 16S rRNA sequences. Though the relative abundance of bacterial phyla differed between nurse and forager gut communities, core taxa were consistent across the worker groups (Fig. 3).

**Table 3.** Relative percentage abundance of 19 bacterial phyla across all workers

Phylum	All workers	Nurses	Foragers
Firmicutes * ‡	46.00	27.08	62.11
Proteobacteria * †	37.65	52.61	24.90
Cyanobacteria *	5.55	6.69	4.58
Bacteroidetes *	4.54	5.99	3.31
Actinobacteria * †	3.86	5.01	2.88
Acidobacteria	0.64	1.15	0.21
Planctomycetes	0.41	0.35	0.47
Gemmatimonadota	0.32	0.26	0.38
Patescibacteria	0.20	0.15	0.24
Deinococcota	0.18	0.08	0.25
Myxococcota	0.17	0.20	0.15
Nitrospirota	0.15	0.16	0.15
Bdellovibrionota	0.13	0.04	0.20
Verrucomicrobia	0.11	0.12	0.11
Spirochaetae	0.02	0.03	0.01
WPS-2	0.02	0.01	0.02
Chloroflexi	0.02	0.03	0.02
Armatimonadota	0.01	0.03	0.00
Desulfobacterota	0.01	0.00	0.01

* Five most abundant core taxa.

† Taxa that appear in greater abundance in forager gut samples.

‡ Taxa that appear in greater abundance in nurse gut samples.

**Fig. 4.**
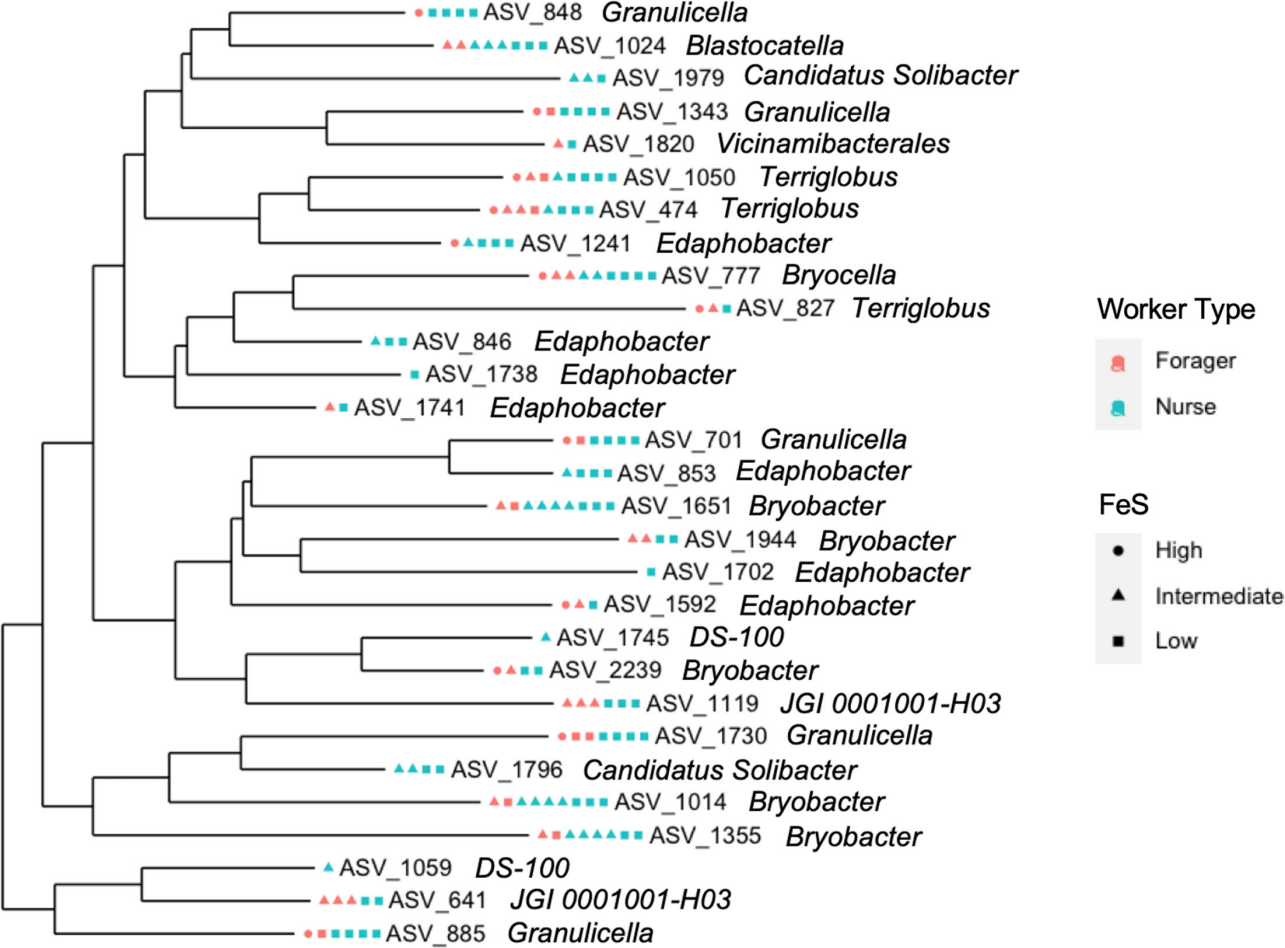
Phylogenetic tree of ASVs classified as Acidobacteria. Each coloured shape represents one sample in which the corresponding ASV was observed.

One prominent difference between worker groups was the abundance of Acidobacteria found in nurses. Though Acidobacteria only comprised 0.64% of taxa across all samples, this phylum appeared in fivefold greater abundance in the gut samples of nurses when compared to those of foragers (Table 3). To further highlight these taxa, a phylogenetic tree was generated to display the distribution of samples among all ASVs belonging to Acidobacteria (Fig. 4). The most abundant genera within this group were *Bryobacter*, *Edaphobacter* and *Terriglobus*.

### Alpha diversity metrics

No significant differences were observed between alpha diversity of gut bacterial communities when grouped by colony or host worker type (Fig. S2), though species belonging to the family Entomoplasmataceae appeared more frequently in association with foragers than with nurses.

**Fig. 5.**
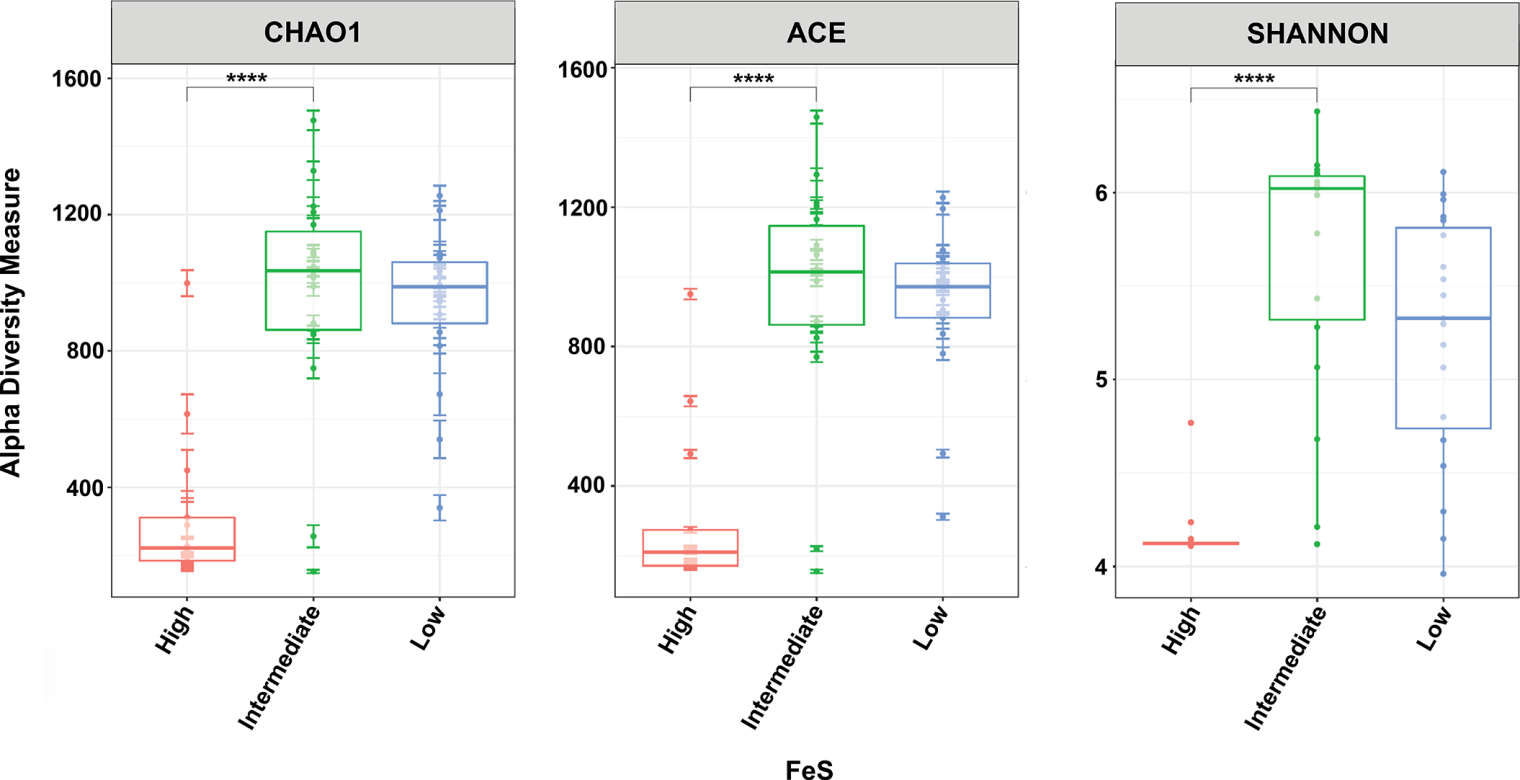
Species richness (Chao1 and ACE) and diversity (Shannon) measurements of bacterial communities grouped by FeS range. Higher Chao1 and ACE indices correspond with greater species richness. Higher Shannon indices correspond with greater species diversity. Asterisks represent significant difference between groups as measured by Kruskal–Wallis tests (*P*<0.0001).

Further, there was no significant effect of intranidal movement or foraging on the abundance or diversity of bacterial communities, though faecal interaction had a significant impact on alpha and beta diversity measurements (Figs5  6). While samples associated with low and intermediate FeSs did not appear to have distinct differences in alpha diversity, samples associated with high FeSs had significantly lower measurements across Chao1, ACE and Shannon indices. Group differences were tested with Kruskal–Wallis tests (*P*<0.0001 for all cases).

**Fig. 6.**
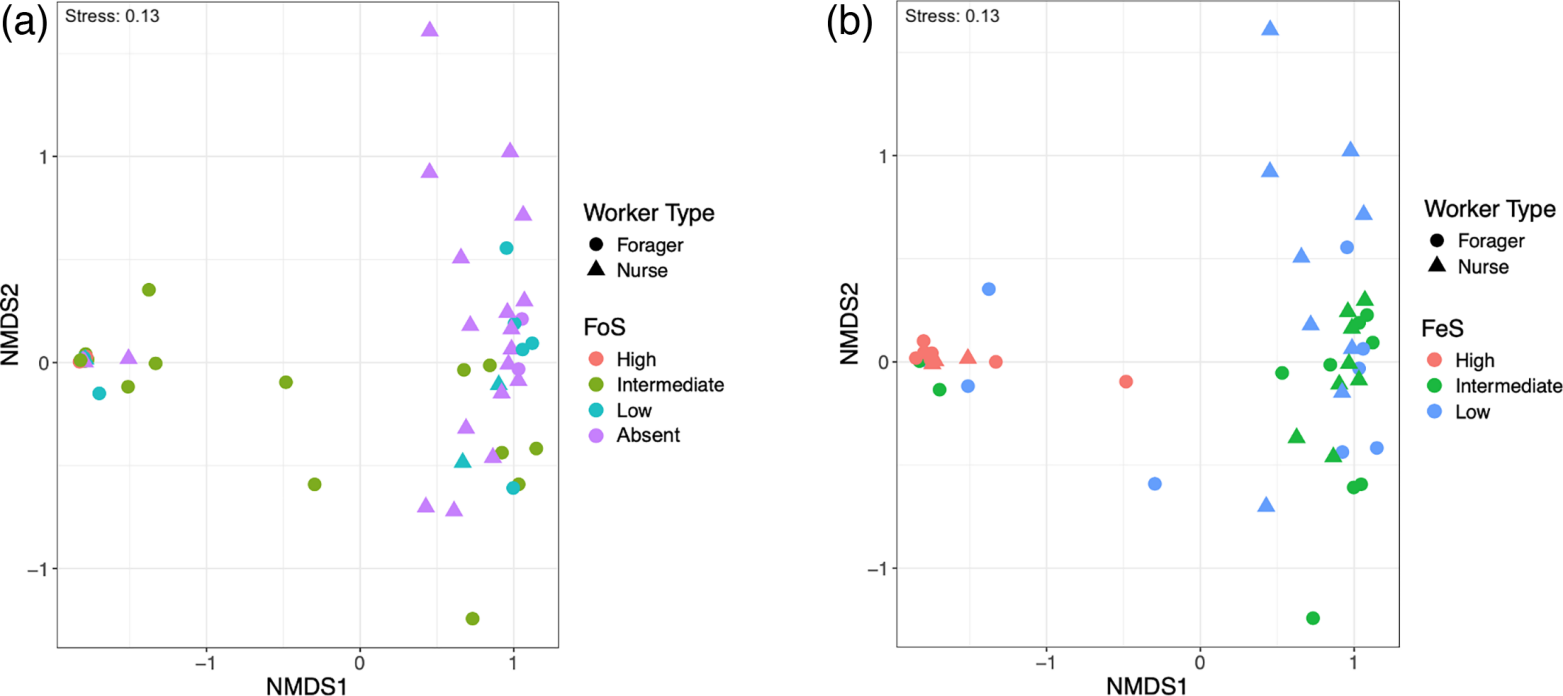
NMDS plots of bacterial communities per sample. Foraging (**a**) and faecal interaction (**b**) levels are represented by each colour.

### Multivariate compositional analysis

NMDS analyses demonstrated that beta diversity of worker gut microbiota differed across samples (Fig. 6). The spatial distribution of ASVs was clustered by worker type and level of faecal interaction rather than by foraging activity, as demonstrated by NMDS (Fig. 6) and confirmed by PERMANOVA (worker type: F.Model=2.5576, *P*<0.05; FeS: F.Model=9.6112, *P*<0.001). Despite variation between colonies, the PERMANOVA test for colony differences did not indicate any statistical significance in activity. The variation observed between different colonies was significantly skewed by *Massilia*, *Entoplasma* and *Blastomonas*, which were among the genera identified by the LEfSe analysis as differentially enriched in samples from colony 48 (Fig. S3). Variation between foragers and nurses was similarly separated by *Sphingomonas*, *Massilia* and *Blastomonas*, with these genera more enriched in samples from nurses (Fig. S4).

**Fig. 7.**
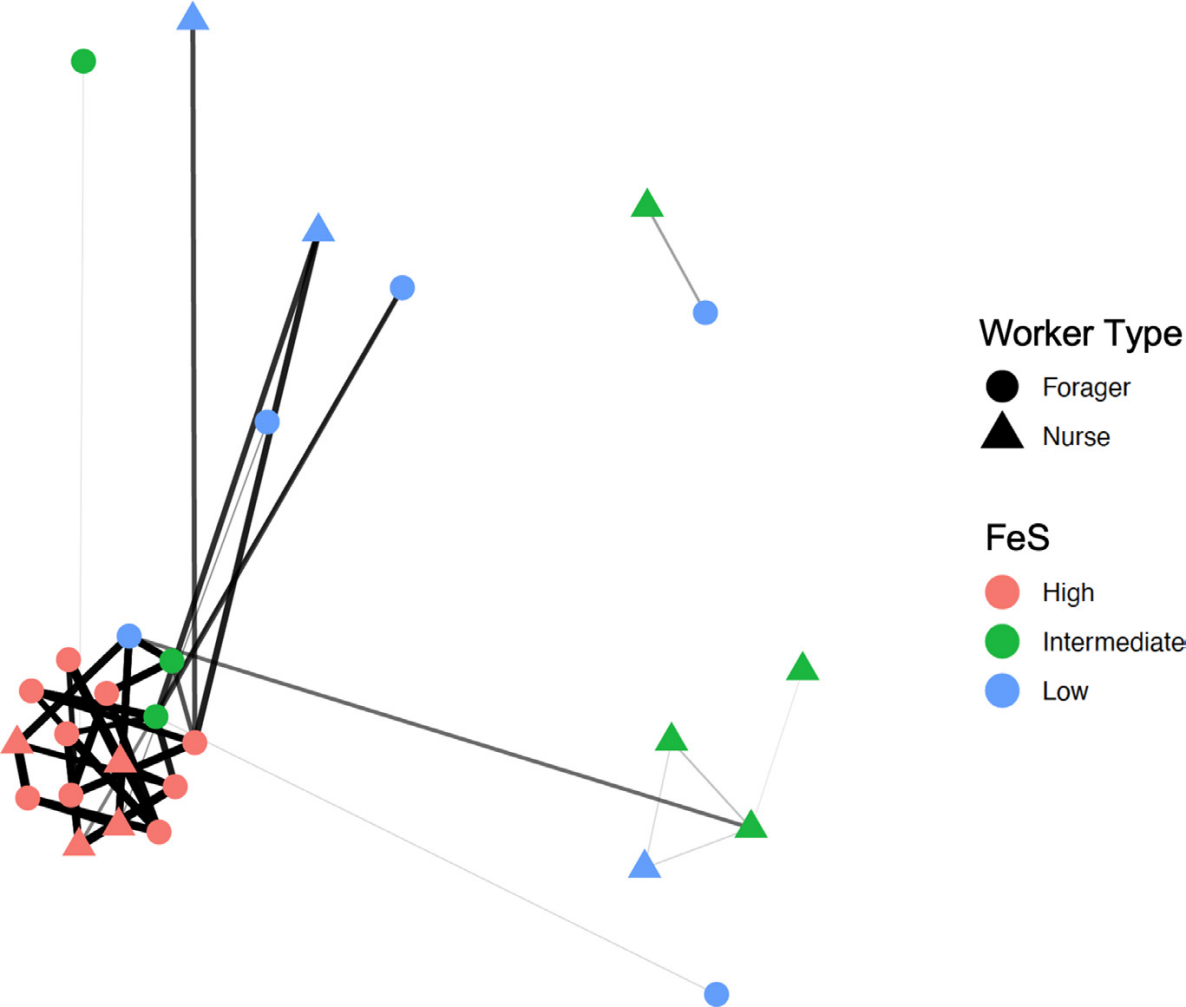
Phylogenetic network analysis of 16S rRNA sequences. Line thickness and length represent inferred taxa co-occurrence within *Aphaenogaster picea* gut microbial communities.

PCoA was performed to visualize dissimilarities between gut bacterial communities as clustered by FeS. Though PCoA plots of unweighted and weighted UniFrac distances demonstrate indiscriminate clustering of low and intermediate FeS samples and tighter clustering of high FeS samples, PERMANOVA did not show statistically significant variance between score groups (*P*=0.389) (Fig. S6). Community clustering and distance between sample groups were further visualized by phylogenetic network analysis, which highlight a co-occurrence pattern of taxa associated with high FeSs (Fig. 7).

## Discussion

Here, we describe the gut microbiota of *Aphaenogaster picea* for the first time. We did not find significant differences in gut microbial communities between castes. We did, however, find that ants that interacted with refuse piles had a bacterial composition distinct from ants that did not. This was largely driven by the number of ASVs detected in the Entomoplasmataceae family.

Analyses of video data did not demonstrate distinct behavioural motifs within each identified task group, aligning with the expectation of weakly defined subcastes within *Aphaenogaster* colonies. No strong relationships between gut bacterial community composition and worker type were immediately evident. This is consistent with the prediction [22] that species with weakly differentiated subcastes will have weakly differentiated microbial communities. Gut bacterial communities were not significantly different between colonies. Analysing microbial taxa diversity in terms of individual faecal interactions with refuse piles revealed certain ASVs that were representative of gut samples from all colonies, worker types and overall tempo (IDS and FoS ranges). Interaction with faecal matter and refuse piles was not behaviourally unique to any particular group. Despite this behaviour spanning all task groups and colonies, only high levels of interaction with refuse piles were strongly associated with distinct bacterial communities (Fig. 5). This has been similarly observed in bumblebees, in which microbiome sharing is common among workers due to interactions with faecal-borne bacteria [60]. Gut bacterial communities grouped by high FeSs were dominated by ASVs associated with Entomoplasmataceae, a bacterial family that appears in many other species of ants, including fungus-growing attines [24,61], weaver ants [37], fire ants [62] and army ants [63]. Entomoplasmataceae belongs to the phylum Firmicutes, which readily appears among gut-colonizing taxa in other insects and mammals [64].

All core taxa (i.e. Firmicutes, Proteobacteria, Cyanobacteria, Bacteroidetes and Actinobacteria) have been previously identified as dominant phyla in soil-dwelling bacterial communities and, as such, are also highly involved in the succession of insect gut environments [21,65, 66]. Seven thousand eight hundred fifty of the 8300 ASVs analysed were classified as chloroplast, suggesting that the detected presence of Cyanobacteria may be an artefact of digested plant materials in the gut. ASVs identified as Firmicutes and Proteobacteria contributed to 83.65% of all relative abundance, with Firmicutes dominating in foragers and Proteobacteria dominating in nurses (Table 3). This difference in relative abundance between gut microbial communities from foragers and nurses may be attributed to differences in degrees of exposure to diet, as nurses inside the nest may not have had as much access to solid and liquid food sources as foragers outside the nest. Nurses that did not leave the nest were limited to diet sources that were brought into the nest by other individuals, though it is possible that nurses were getting regurgitated food from larvae. Other insect gut microbiome studies have reported higher abundances of Proteobacteria and Firmicutes in herbivorous and omnivorous hosts, respectively, suggesting that it is perhaps diet availability between subcastes that drives differences in core microbiota abundance [37,67, 68]. For foragers and generalists that had concurrently high FoS and FeS, diets may have been more significantly composed of protein-rich matter from refuse piles, while carbohydrate-rich food brought into the nest was reserved for nurses and larvae [31]. However, it is interesting to consider the absence of families such as Acetobacteraceae and Lactobacillaceae taxa that typically dominate the gut environments of omnivorous arthropods and mammals [39,68]. We identified ASVs belonging to the family Rickettsiaceae but did not specifically detect any *Wolbachia* species, common endosymbionts found in ants and other arthropods [69,70]. The reduced abundance of these taxa may further emphasize the role of faecal interactions in shaping the microbiota of *Aphaenogaster picea* by introducing taxa capable of outcompeting established gut symbionts.

Clustering patterns of samples grouped by FeSs (Figs 6b and S6) are largely consistent with diet-oriented microbiome studies reporting synergies between low alpha diversity and high beta diversity [71,72]. For example, a study of pika (mammal) diets found that more diverse diets do not always correlate with diverse gut microbiota contributing to higher species richness, but diets of similar composition do correlate with more similar gut microbiota [71]. When considering faecal interaction as a proxy for diet, a similar effect is observed between the gut microbiota of individuals with more ‘diverse’ FeSs (i.e. low to intermediate) and that of individuals with more similar FeSs (i.e. high). Phylogenetic network analysis of ASVs demonstrated that gut bacterial samples from ants with high FeSs, independent of worker type, cluster closely together (Fig. 7). The short distances between samples with high FeS, independent of worker type, emphasize that faecal interactions were the most important factor in shaping bacterial communities. There is little discernible difference between samples with low and intermediate FeSs, perhaps also suggesting that bacteria associated with refuse piles and faecal matter might be adept at outcompeting pre-existing taxa.

ASVs belonging to the phylum Acidobacteria were identified in slightly greater abundance in the gut samples of nurses (Fig. 4). The most abundant genera of Acidobacteria found across all samples were *Bryobacter*, *Edaphobacter* and *Terriglobus*, which, like many members of this phylum, are largely found in soil samples, in low pH conditions and in association with insects [65,73]. Acidobacteria have also been observed in close affiliation with Proteobacteria, which is represented in this study by similarities in percentage abundance across all groups (Table 3). This association may be linked to a high involvement of Acidobacteria in carbon and nitrogen metabolism, functionally paralleling the importance of Proteobacteria in insect gut communities [65]. The gut environment of nurses may be more selective for Acidobacteria, Proteobacteria, Bacteroidetes and Actinobacteria in order to assist in overall brood care, as these phyla all provide a host with enzymes capable of augmenting immune responses [22,28]. Enzymes that assist in digestion of diet nutrients (e.g. Proteobacteria, Acidobacteria and Bacteroidetes), nitrogen metabolism (e.g. Proteobacteria and Acidobacteria) and biosynthesis of essential aas and vitamins (e.g. Proteobacteria, Bacteroidetes and Actinobacteria) would be beneficial towards nursing behaviour, facilitating increased larval survival and increased overall colony fitness [1,4, 22]. In this regard, Acidobacteria are particularly important for synthesizing secondary metabolites such as antifungal and antibiotic agents [65].

*Aphaenogaster picea* notably does not engage in oral trophallaxis, which may explain differences in relative taxa abundance when compared to that of other ant microbiome studies. In recent publications, oral trophallaxis has explained the existence of shared microbial communities between subcastes. This has been widely studied in *Cephalotes*, a genus in which the regurgitation of food from foragers to other workers maintains a colony-wide microbiome [33,34]. Rather than mouth-to-mouth exchange of liquid food, *Aphaenogaster picea* performs tool-assisted food transport by dropping debris into liquid food and carrying the soaked debris into the nest [74,75]. This was observed in video data showing a high incidence of individuals visiting liquid diet sources immediately after interacting with solid diet or refuse piles and may explain the transmission of faecal-borne bacteria to nurses that did not leave the nest. This tool use behaviour is likely what led to the accumulation of refuse piles inside the nest.

After observing *Temnothorax* ants, Segers *et al*. [25] concluded that microbial community abundance did not differ between reproductive and behavioural castes. Though this conclusion aligns with the results of the current study, there are some reasons to suspect that these results do not provide a full picture of the relationship between microbial diversity and division of labour. Given that we, like Segers *et al*. [25], extracted DNA from whole abdomens, differences in bacterial diversity among different functional compartments in the gut may have been overlooked [33,34, 76]. Due to the size of *Aphaenogaster picea*, it was difficult to isolate individual gut compartments; as such, differences in bacterial abundance and diversity within each gut compartment due to pH and proventricular filtering cannot be resolved from data obtained here. Additionally, in smaller colonies, microbiome-linked subcastes have been attributed to temporal modes of division of labour, in which younger workers perform intranidal tasks and older workers perform foraging tasks, leading to different microbial communities that are reflective of the different environments of each subcaste [6,66]; however, we did not collect data on age-related differences in microbiota in this study.

Sequencing results from this study may be useful in informing subsequent microbial work with *Aphaenogaster picea*. It should be noted that sequencing data contained considerable chloroplast abundance; previous studies demonstrated that high levels of plastid contamination may be common in herbivorous insect samples [77]; however, further work should aim to reduce such presence of non-target reads. In the future, it would be salient to cultivate and sequence any observable bacterial or fungal growth in the nest in order to survey the innate microbial community composition from the source habitat. Additional work should be done to understand the role of the microbiota in shaping colony productivity and fitness, which would require sampling larvae, increasing sample size and co-investigating closely related species (e.g. *A. rudis*).

## supplementary material

10.1099/acmi.0.000832.v4 Uncited Supplementary Material 1.

## Abbreviations

ACE: abundance coverage estimator
ASV: amplicon sequence variant
%E: % extranidal
FeS: faecal interaction score
FoS: foraging score
IMS: intranidal movement score
LEfSe: linear discriminant analysis effect size
NMDS: non-metric multidimensional scaling
PCoA: principal coordinate analysis
PERMANOVA: permutational multivariate ANOVA
SRA: sequence read archive
