# Supplementary material for: Interaction with refuse piles is associated with co-occurrence of core gut microbiota in workers of the ant Aphaenogaster picea
Source: Access Microbiol. 2025 Jan 30;7(1):000832.v4. doi: 10.1099/acmi.0.000832.v4 (PMC11781628; doi:10.1099/acmi.0.000832.v4)
Supplement: Uncited Supplementary Material 1. [file acmi-7-00832-s001.pdf]

Table S1: Activity counts per individual worker. % Extranidal (%E) scores were calculated by dividing the amount of time each individual spent outside of the nest by the total time observed and multiplying the score by 100%. Intranidal movement score (IMS), foraging score (FoS), and fecal interaction score (FeS) were each calculated as the number of instances of that behavior divided by the total time observed per worker.

SRA Accession #	Sample ID	Worker Type	Total Time Observed (s)	%E	IMS	FoS	FeS
SRS20462176	A48-F1-G	Forager	891	80.9	19.82	0.51	0.51
SRS20462177	A48-F10-G	Forager	600	94.5	0.00	0.00	0.00
SRS20462188	A48-F2-G	Forager	1034	76.1	10.33	0.31	0.43
SRS20462199	A48-F3-G-a	Forager	1101	100.0	0.00	0.45	0.25
SRS20462210	A48-F3-G-b	Forager	971	90.8	9.55	0.96	0.22
SRS20462221	A48-F4-G-a	Generalist	437	63.2	1.55	0.23	0.11
SRS20462223	A48-F4-G-b	Forager	1103	82.5	2.86	0.38	0.96
SRS20462224	A48-F5-G	Forager	608	100.0	0.00	0.08	0.16
SRS20462225	A48-F6-G	Forager	822	87.1	8.03	0.00	1.23
SRS20462226	A48-F7-G	Generalist	895	32.5	5.86	0.12	0.95
SRS20462178	A48-F8-G	Forager	429	100.0	0.00	0.12	0.47
SRS20462179	A48-F9-G	Forager	785	100.0	0.00	0.48	0.37
SRS20462180	A48-N10-G	Nurse	1200	0.0	3.92	0.00	0.00
SRS20462181	A48-N3-G	Nurse	555	24.3	5.48	0.00	0.18
SRS20462182	A48-N4-G	Nurse	1028	0.0	0.92	0.00	0.00
SRS20462183	A48-N5-G	Nurse	560	8.6	4.79	0.00	0.18
SRS20462184	A48-N6-G	Nurse	564	0.0	0.71	0.00	0.00
SRS20462185	A48-N7-G	Nurse	867	9.1	5.63	0.00	0.44

SRS20462186	A48-N8-G	Nurse	992	21.4	6.60	0.00	0.67
SRS20462187	A48-N9-G	Nurse	1080	0.0	0.17	0.00	0.00
SRS20462189	A51-F10-G	Forager	1200	79.1	1.36	0.50	0.16
SRS20462190	A51-F2-G	Forager	596	81.7	9.17	0.25	0.00
SRS20462191	A51-F3-G	Forager	1173	100.0	0.00	0.60	0.00
SRS20462192	A51-F8-G	Forager	889	94.7	0.00	0.34	0.17
SRS20462193	A51-F9-G	Generalist	1011	67.1	4.66	0.08	0.24
SRS20462194	A51-N1-G	Nurse	1247	11.1	4.87	0.08	0.00
SRS20462195	A51-N2-G	Nurse	980	0.0	1.73	0.00	0.13
SRS20462196	A51-N3-G	Nurse	1198	3.8	4.76	0.00	0.00
SRS20462197	A51-N5-G	Nurse	1060	9.8	16.09	0.00	0.41
SRS20462198	A51-N6-G	Generalist	1243	28.8	7.08	0.00	0.16
SRS20462200	A53-F1-G	Forager	1175	95.8	37.78	1.33	0.34
SRS20462201	A53-F10-G	Generalist	1152	65.8	6.82	0.74	1.30
SRS20462202	A53-F2-G	Forager	924	100.0	0.00	0.54	0.00
SRS20462203	A53-F3-G	Forager	946	82.2	6.62	0.48	0.80
SRS20462204	A53-F4-G	Forager	422	100.0	0.00	0.71	0.12
SRS20462205	A53-F5-G	Forager	1071	100.0	0.00	0.60	0.51
SRS20462206	A53-F6-G	Forager	600	100.0	0.00	0.33	0.00
SRS20462207	A53-F7-G	Forager	995	100.0	0.00	0.46	0.99
SRS20462208	A53-F8-G	Forager	876	100.0	0.00	0.43	0.09
SRS20462209	A53-F9-G	Generalist	601	73.0	14.20	0.17	0.25

SRS20462211	A53-N1-G	Nurse	563	0.0	4.97	0.00	0.36
SRS20462212	A53-N10-G	Nurse	600	0.0	2.08	0.00	0.00
SRS20462213	A53-N2-G	Nurse	1166	21.5	15.48	0.18	0.43
SRS20462214	A53-N3-G	Nurse	1005	0.0	0.83	0.00	0.00
SRS20462215	A53-N4-G	Nurse	858	0.0	2.34	0.00	0.42
SRS20462216	A53-N5-G	Nurse	600	0.0	0.25	0.00	0.00
SRS20462217	A53-N6-G	Nurse	988	0.0	0.38	0.00	0.00
SRS20462218	A53-N7-G	Nurse	810	0.0	0.71	0.00	0.00
SRS20462219	A53-N8-G	Nurse	600	0.0	4.25	0.00	0.58
SRS20462220	A53-N9-G	Nurse	1002	21.4	16.88	0.09	1.48
SRS20462222	Control	--	--	--	--	--	--

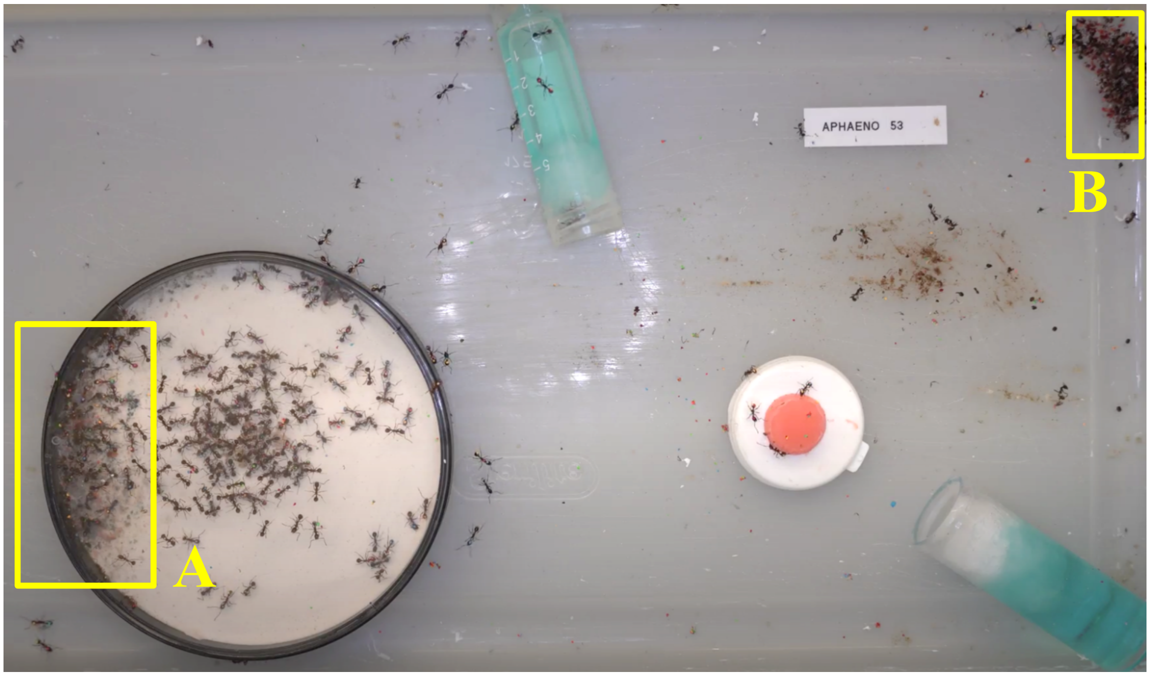

Figure S1: Sample still taken from video data showing refuse piles inside (a) and outside (b) of the nest.

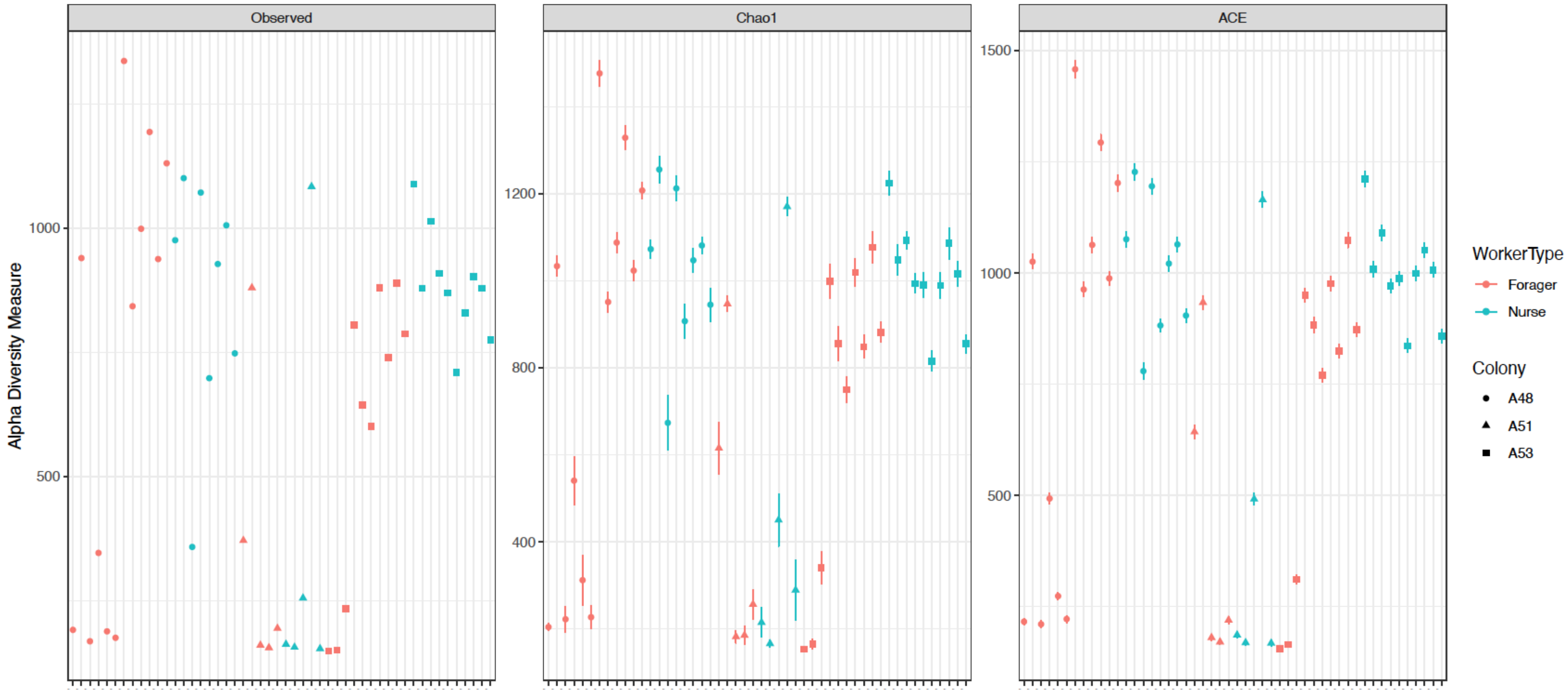

Figure S2: Alpha diversity measurements of bacterial communities per *A. picea* gut sample.

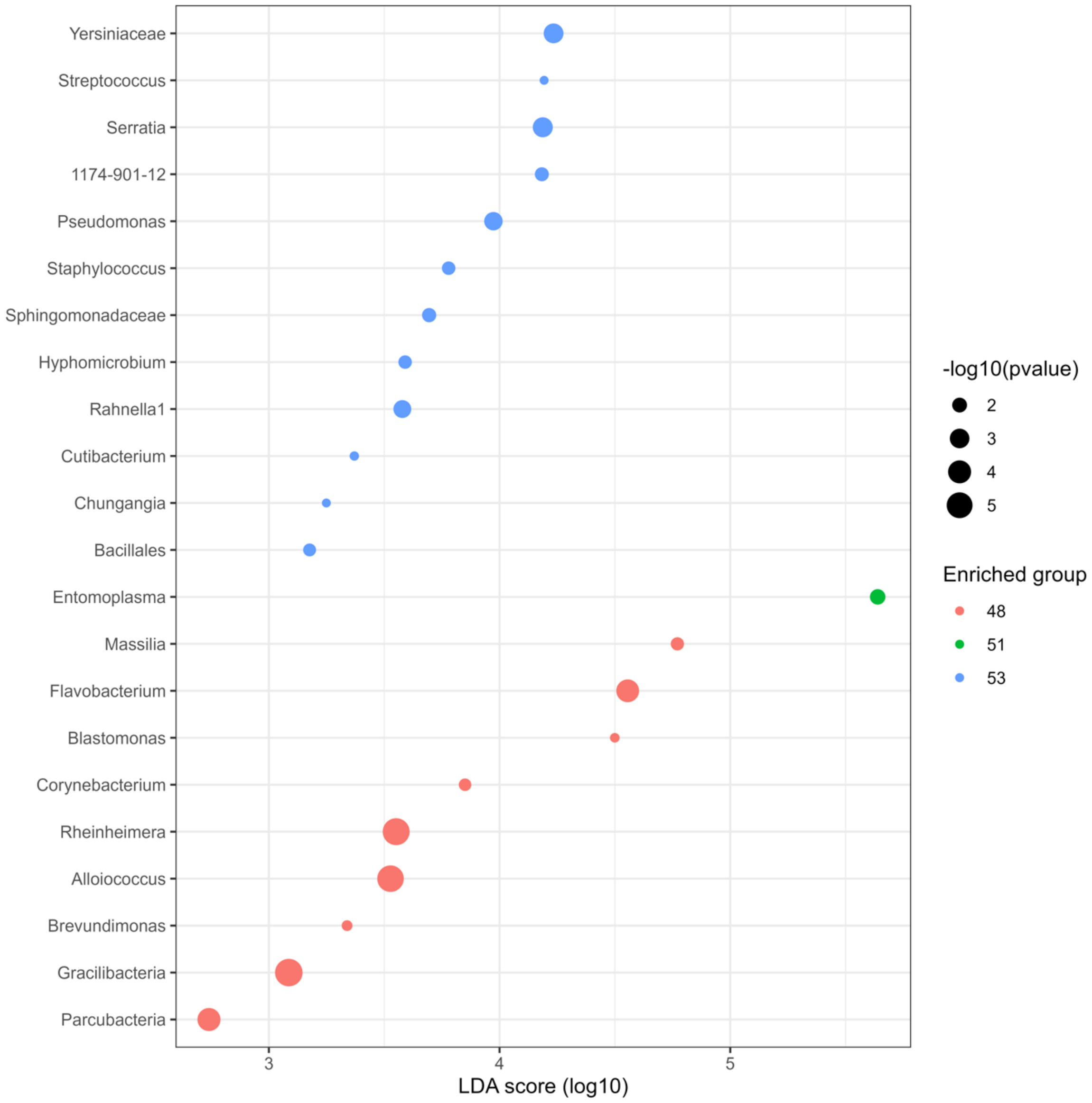

Figure S3: Logarithmic Linear Discriminant Analysis (LDA) scores to estimate effect size of each differentially abundant species per colony.

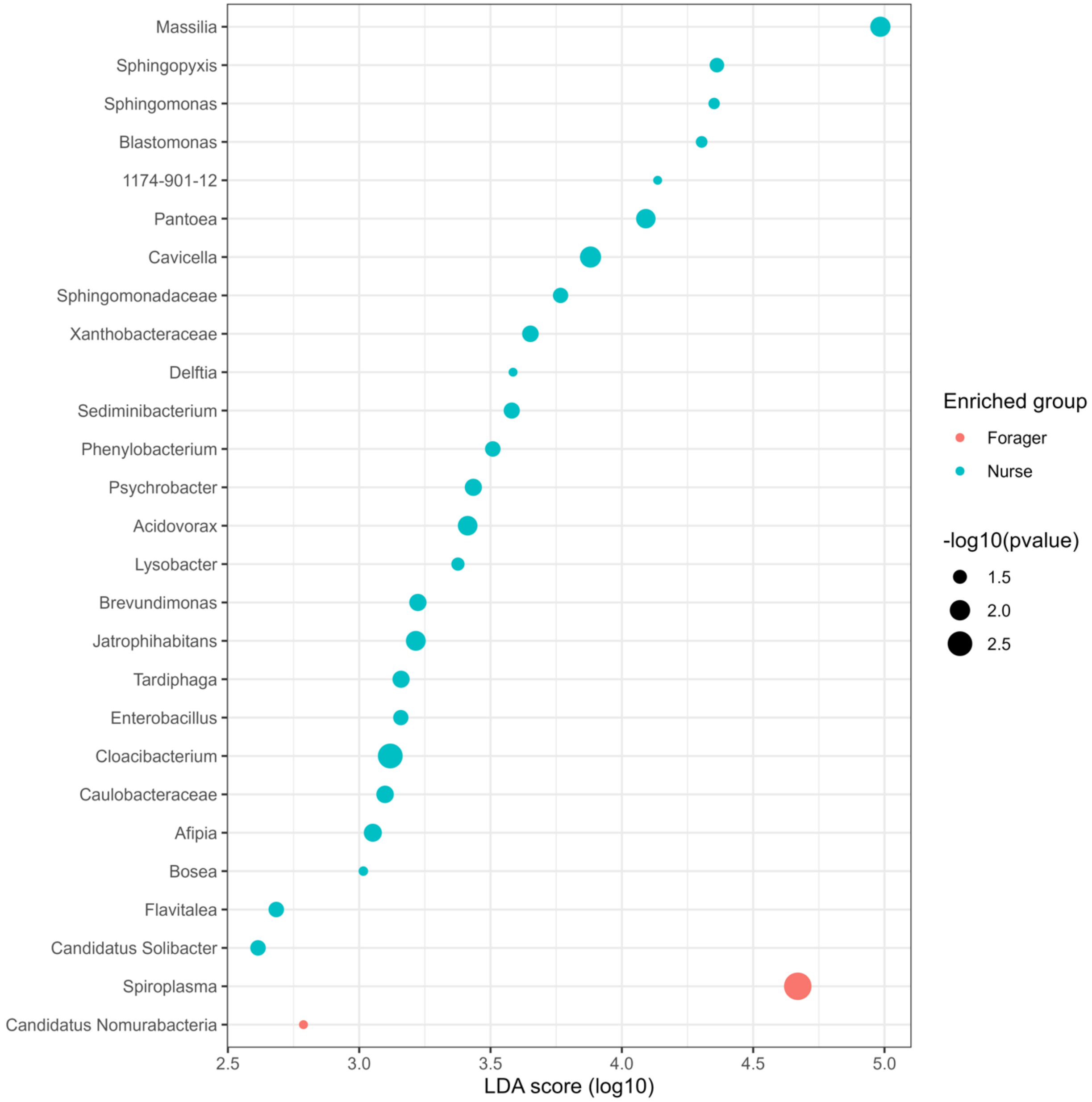

Figure S4: Logarithmic Linear Discriminant Analysis (LDA) scores to estimate effect size of each differentially abundant species per worker type.

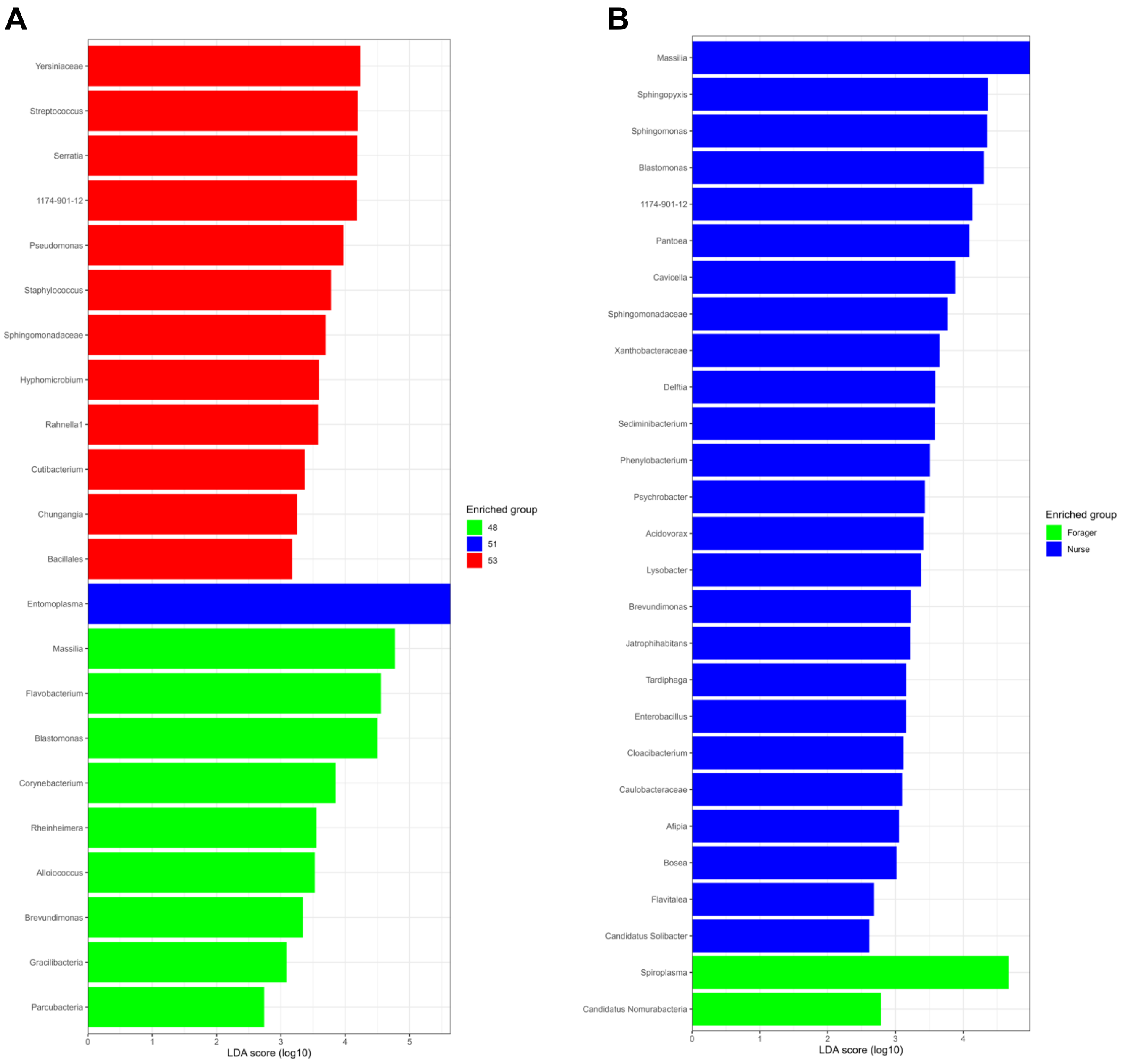

Figure S5: Histogram of logarithmic Linear Discriminant Analysis (LDA) scores to estimate effect size of each differentially abundant species per colony **(A)** and worker group.

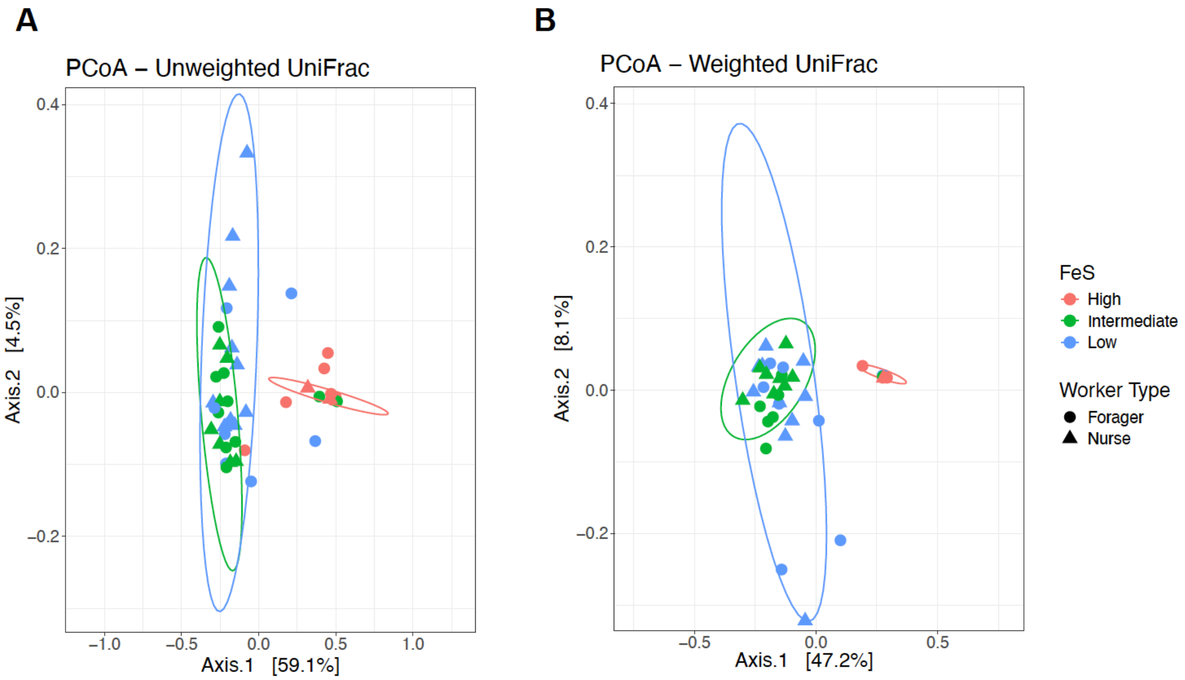

Figure S6. Gut bacterial communities of workers clustered by principal coordinate analysis (PCoA) based on unweighted (a) and weighted (b) UniFrac distances. Worker types are represented by shape and fecal interaction score (FeS) ranges are represented by color. The axes represent the percentage contribution of the principal coordinate component to composition differences between samples.

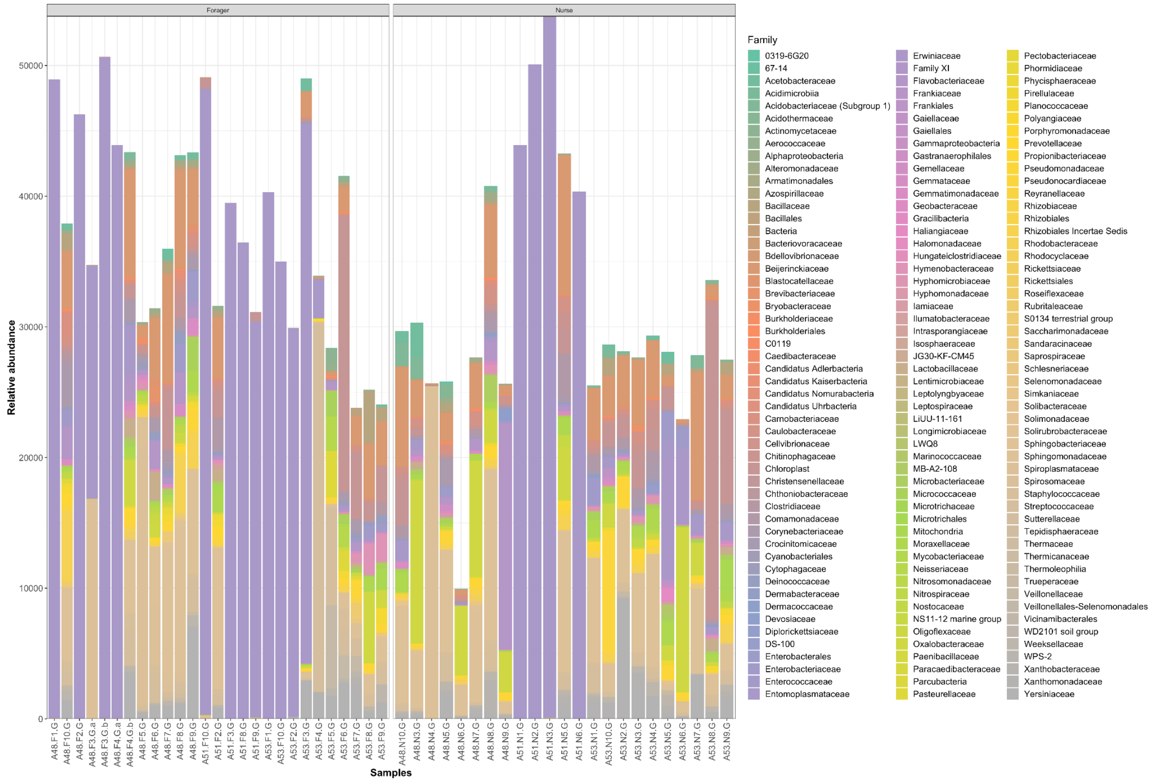

Figure S7: Relative abundance of bacterial families per gut sample community. The plot is split into two subplots, showing the abundance of taxa in foraging (left) and nurse (right) ants. In this analysis, generalists with %Extranidal scores less than 50% were considered nurses and generalists with %Extranidal scores greater than 50% were considered foragers (Table S1).

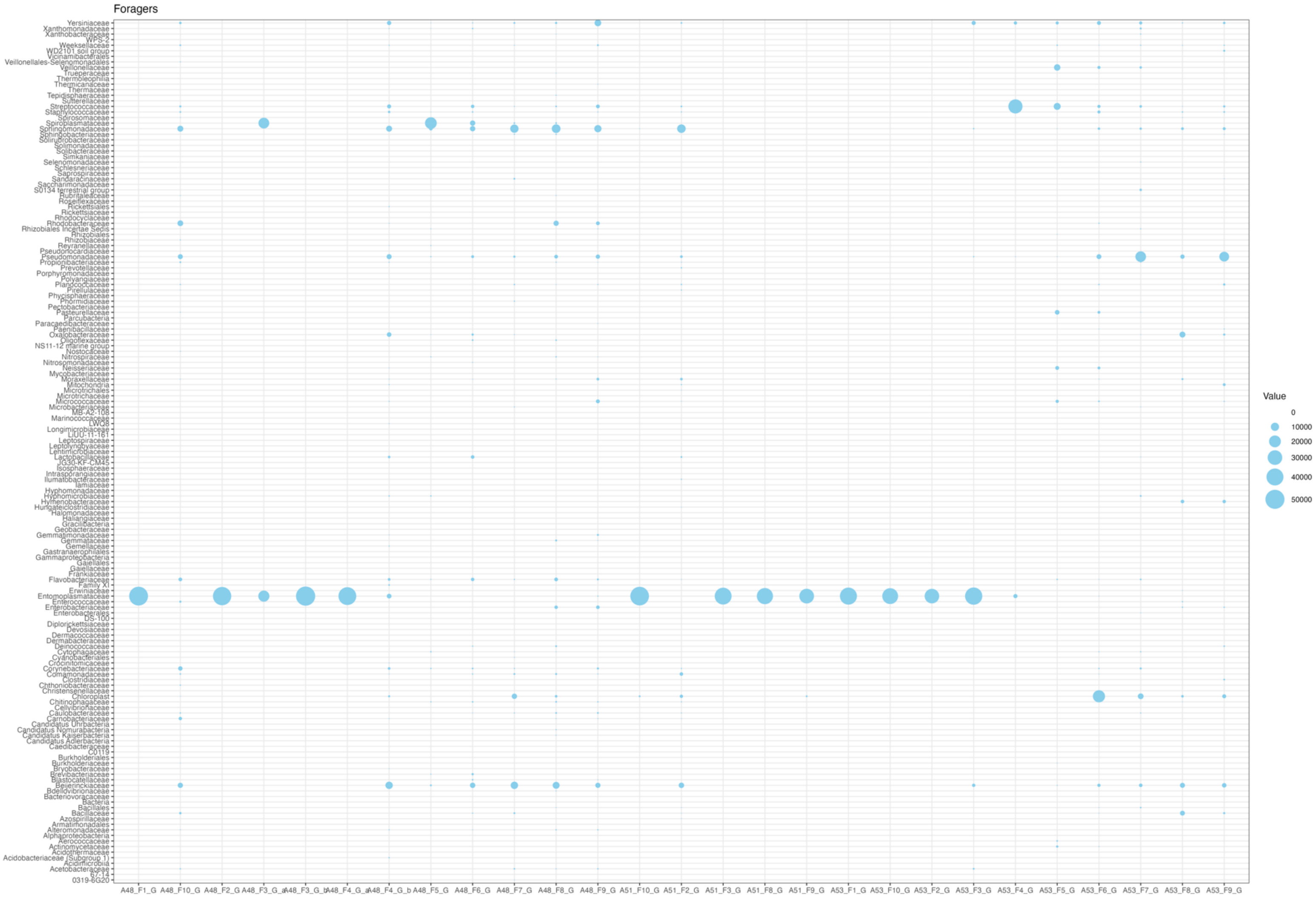

Figure S8: Ballon plot of relative abundance of bacterial families per forager gut sample.

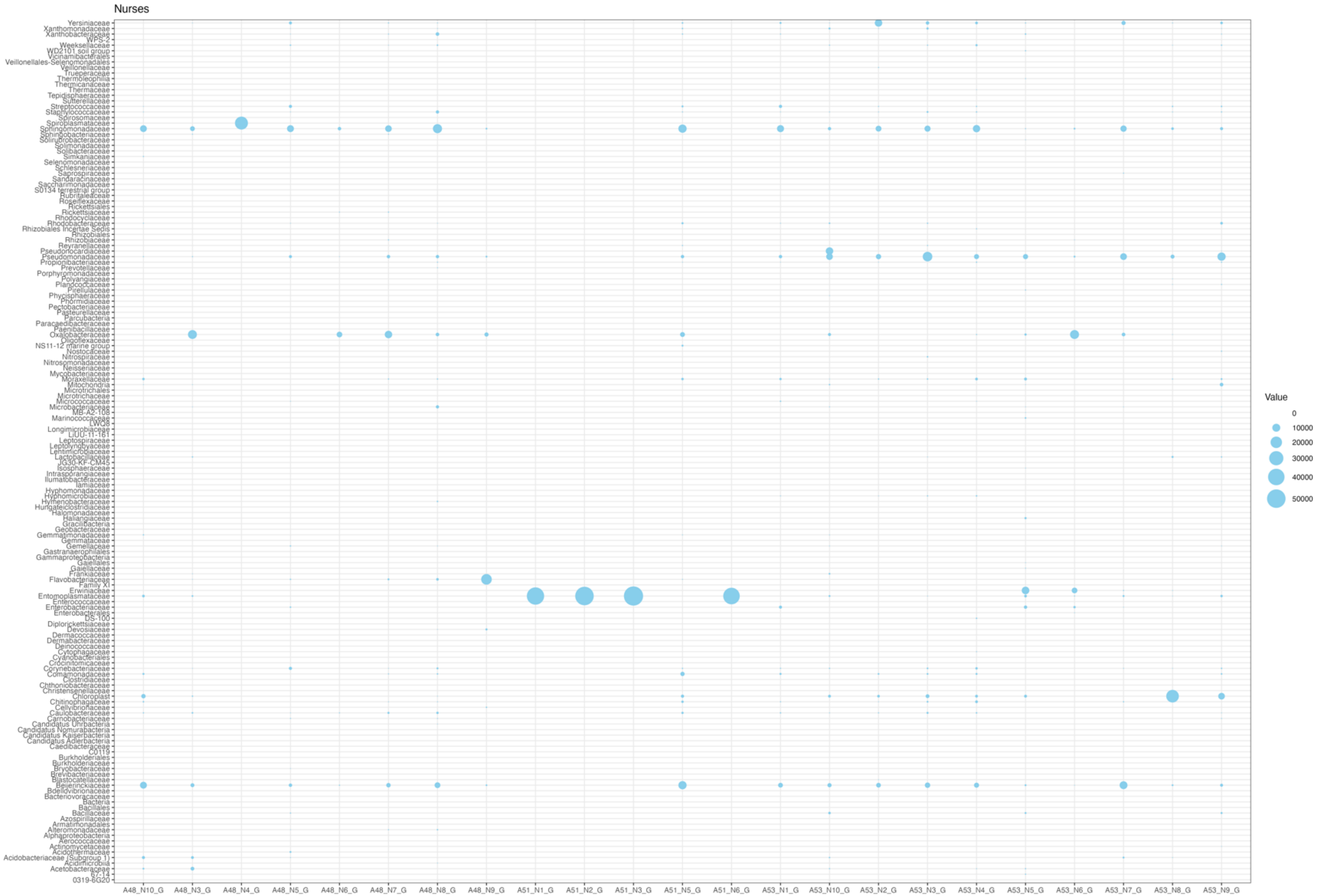

Figure S9: Ballon plot of relative abundance of bacterial families per nurse gut sample.
